# Visualizing the Attack of RNase Enzymes on Dendriplexes and Naked RNA Using Atomic Force Microscopy

**DOI:** 10.1371/journal.pone.0061710

**Published:** 2013-04-18

**Authors:** Hosam G. Abdelhady, Yen-Ling Lin, Haiping Sun, Mohamed E. H. ElSayed

**Affiliations:** 1 College of Pharmacy, Taibah University, Al Madinah Al Munawwarah, Saudi Arabia; 2 National Organization for drug control and Research, Cairo, Egypt; 3 Department of Biomedical Engineering, University of Michigan, Cellular Engineering and Nano-Therapeutics Laboratory, Ann Arbor, Michigan, United States of America; 4 Department of Materials Science and Engineering, University of Michigan, Electron Microbeam Analysis Laboratory, Ann Arbor, Michigan, United States of America; 5 Macromolecular Science and Engineering Program, University of Michigan, Ann Arbor, Michigan, United States of America; McGill University, Canada

## Abstract

Cationic polymers such as poly(amidoamine), PAMAM, dendrimers have been used to electrostatically complex siRNA molecules forming dendriplexes for enhancing the cytoplasmic delivery of the encapsulated cargo. However, excess PAMAM dendrimers is typically used to protect the loaded siRNA against enzymatic attack, which results in systemic toxicity that hinders the *in vivo* use of these particles. In this paper, we evaluate the ability of G4 (flexible) and G5 (rigid) dendrimers to complex model siRNA molecules at low +/− ratio of 2/1 upon incubation for 20 minutes and 24 hours. We examine the ability of the formed G4 and G5 dendriplexes to shield the loaded siRNA molecules and protect them from degradation by RNase V1 enzymes using atomic force microscopy (AFM). Results show that G4 and G5 dendrimers form similar hexagonal complexes upon incubation with siRNA molecules for 20 minutes with average full width of 43±19.3 nm and 62±8.3 at half the maximum height, respectively. AFM images show that these G4 and G5 dendriplexes were attacked by RNase V1 enzyme leading to degradation of the exposed RNA molecules that increased with the increase in incubation time. In comparison, incubating G4 and G5 dendrimers with siRNA for 24 hours led to the formation of large particles with average full width of 263±60 nm and 48.3±2.5 nm at half the maximum height, respectively. Both G4 and G5 dendriplexes had a dense central core that proved to shield the loaded RNA molecules from enzymatic attack for up to 60 minutes. These results show the feasibility of formulating G4 and G5 dendriplexes at a low N/P (+/−) ratio that can resist degradation by RNase enzymes, which reduces the risk of inducing non-specific toxicity when used *in vivo*.

## Introduction

Preclinical investigations showed the potential of small interfering RNA (siRNA) molecules in selectively silencing the expression of the genes implicated in the development of cancer, cardiovascular, neurodegenerative, and infectious diseases indicating their therapeutic potential [1], [2], [3], [4]. siRNA molecules bind to the RNA-induced silencing complex (RISC) revealing the antisense RNA strand that selectively binds to the complementary sequence in the targeted mRNA, which triggers mRNA cleavage by the endonuclease RNase H enzymes and suppression of the translation process [5], [6], [7]. Delivery of siRNA molecules requires a biocompatible carrier to protect and shuttle the cargo into the cytoplasm of the diseased cells to produce the desired therapeutic activity both *in vitro* and *in vivo*. Many cationic peptides, lipids, and polymers have been used to condense siRNA via electrostatic interaction forming ionic complexes with variable size and surface charge, which proved effective in delivering the RNA cargo into the cytoplasm of mammalian cells *in vitro* [8], [9], [10], [11], [12]. However, successful *in vivo* delivery of siRNA required the use of excess cationic carrier to shield and protect the RNA cargo against nucleases leading to non-specific distribution of the formed complexes to the reticular endothelial system (liver, spleen, and bone marrow) [13] and induction of toxicity [14], which hampered the translation of these particles into the clinic.

Poly(amidoamine), PAMAM, dendrimers are a family of water-soluble polymers that is characterized by a unique, highly-ordered, three dimensional, tree-like branching architecture with a large number of primary, secondary, and tertiary amine groups embedded in their structure, which become ionized at physiologic pH conferring a high positive charge density [15], [16]. PAMAM dendrimers show a controlled incremental increase in the size, molecular weight, and number of surface amine groups with the increase in their generation number (G). Steric crowding of the surface groups affects the molecular shape of PAMAM dendrimers where G0-G4 adopt an open planar and elliptical conformation whereas higher generations (≥G5) are robust, non-deformable, spheroids [17], [18]. PAMAM dendrimers have been used to complex plasmid DNA (pDNA), antisense oligonucleotides (ASODN), and siRNA molecules into compact nanoparticles that proved to successfully escape the endosomal/lysosomal trafficking pathway through their endosomal buffering capacity known as the “proton sponge” mechanism [19], [20], [21]. However, stabilization of nucleic acid cargo and successful intracellular delivery requires the use of high PAMAM dendrimer (+) to nucleic acid (−) ratio [22], which is often associated with destabilization of the cell membrane and non-specific toxicity [23]. We are interested in formulation of compact dendriplexes that resist degradation by RNase enzymes without using excess PAMAM dendrimers to eliminate the associated toxicity.

Earlier studies showed that DNA condensation has two kinetic phases starting with an initial rapid binding (within 15 seconds) of DNA to multivalent cations followed by slower structural rearrangement that reaches an apparent equilibrium typically within 1–2 hours and exhibit insignificant changes at longer incubation times [24]. The effect of incubation time of cationic PAMAM dendrimers with pDNA molecules on the morphology and stability of the formed dendriplexes has been reported [25]. Briefly, incubation of G4 dendrimers with pDNA for 15 minutes resulted in formation of incomplete toroidal complexes or multimeric intermediates that resisted degradation by DNase I enzymes for 1 hour [25]. In comparison, increasing the incubation time of G4 dendrimers with pDNA to 2 hours resulted in the formation of ring-like toroidal complexes that resisted degradation by DNase I enzymes for up to 10 hours [25]. These results indicate that increasing the incubation time of cationic PAMAM dendrimers with pDNA results in formation of more compact particles that better shield the complexed DNA molecules and protect them against degradation by nuclease enzymes.

It is important to note that pDNA molecules exist in solution as long flexible chains with an average length of ∼1.2 µm, which allow them to wrap around cationic carriers forming compact particles that resist degradation by DNase enzymes and achieve high transfection [14], [26]. In comparison, siRNA molecules are much shorter (∼6 nm) rigid rods in solution that exhibit weak electrostatic interaction with cationic carrier, which increases their susceptibility to enzymatic attack, reduces their internalization by mammalian cells, and diminishes their net transfection [14], [26]. Further, flexibility of the cationic carrier play a critical role in governing its electrostatic binding to siRNA molecules [27]. Previous computational studies showed that flexible cationic carriers assume a spherical shape with a compact core and strong orientation of the cationic surface groups toward the polynucleotides (e.g. DNA, RNA) present in solution forming individual binding points characterized by high binding strength [27]. On the other hand, rigid cationic carriers form more contact points with polynucleotides present in solution [27]. These studies collectively show that both carrier flexibility and incubation time affect the morphology and enzymatic stability of the formed complexes.

In this article, we describe the complexation of anti-GAPDH siRNA molecules with G4 (flexible) and G5 (rigid) dendrimers based on the size and morphology of the formed dendriplexes at different incubation times (20 minutes and 24 hours). We also investigate the stability of the formed dendriplexes upon incubation with RNase V1 enzymes compared to naked siRNA molecules as a function of exposure time. We used atomic force microscopy (AFM) to visualize the morphology of the formed complexes and monitor the attack of RNase V1 enzymes in solution as a function of time. We relied on established AFM protocols used to study the dynamics of DNA binding to PAMAM dendrimers [25], [28], polyethylenimine [29], and polylysine [30] forming nanoparticles with different morphologies. AFM has also been used to investigate the degradation of free DNA by endonuclease and exonuclease enzymes in solution [31], [32], which supports our study.

## Materials and Methods

### Materials

G4 (formula weight 14,215 Da) and G5 (formula weight 28,826 Da) with ethylene diamine cores were purchased from Dendritic Nanotechnologies, Inc. (Mount Pleasant, MI) as 10% w/v solutions in methanol. G4 and G5 solutions were dialyzed using Slide-A-Lyzer dialysis cassettes with a 7 kDa MWCO (Thermo Scientific Inc., Rockford, IL) against DI water for 24 hours to remove polymer debris. The aqueous solutions of G4 and G5 were lyophilized and stored at 4°C till used. Anti-GAPDH siRNA and RNase V1 enzyme were purchased from Ambion Inc. (Austin, TX).

### Formulation of dendriplexes

G4 and G5 dendrimers were dissolved in RNase-free water and mixed with 0.7 µg of anti-GAPDH siRNA molecules dissolved in 1 µl of RNase-free water at a nitrogen/phosphate (N/P, +/−) ratio of 2/1. Each mixture was vortexed and allowed to stand at room temperature for 20 minutes or 24 hours before loading onto a 1% w/v agarose gel containing ethidium bromide (EtBr). The gel was immersed in a Tris-acetate-EDTA (TAE) buffer and run at 60 V for 1 hour and visualized under UV exposure (Fotodyne Incorporated, Hartland, WI).

### AFM imaging of dendriplexes and naked siRNA

All AFM images were acquired using Nanoscope III MultiMode AFM with a sharp nitride lever (Veeco, Santa Barbra, CA) in the tapping mode at a 256×256 pixel resolution. Selected fields were scanned at scan rates ranging from 3–4 Hz where each image was acquired within 90 seconds and tapping frequencies ranged from 8–10.5 kHz in solution. Images were flattened to account for Z offsets and sample tilts. Tapping set points were selected close to the free oscillation amplitude to reduce forces exerted on the interfacial species. All imaging experiments started with scanning the mica substrate in DI water to confirm the absence of any adsorbed contaminants. Naked anti-GAPDH siRNA was imaged using 30 µl of siRNA solution (3.92 µg/ml) in 1 mM PBS containing 2 mM MgCl_2_ where MgCl_2_ ions would allow weak adsorption of anionic siRNA molecules to the negatively charged mica surface through electrostatic interaction following established protocols [33]. Similarly, 30 µl of G4 and G5 dendriplexes were added to freshly cleaved mica and covered with the liquid cell. A 20 µl aliquot of the solution in the liquid cell was replaced with 20 µl of RNase V1 enzyme (6.6 U/ml) diluted with 1 mM PBS of pH 7.4 to examine the effect of RNase enzyme on free siRNA and the dendriplexes. Imaging of naked siRNA and the dendriplexes started immediately after adding their solutions to mica surface with continuous recording for at least 5 minutes after visualizing each subject to allow thorough investigation of the morphology before the treatment with RNase V1 enzymes. Naked siRNA and dendriplexes were also imaged after the addition of RNase V1 enzymes as a function of time.

## Results

### Formulation of G4 and G5 dendriplexes

**Figure 1:**
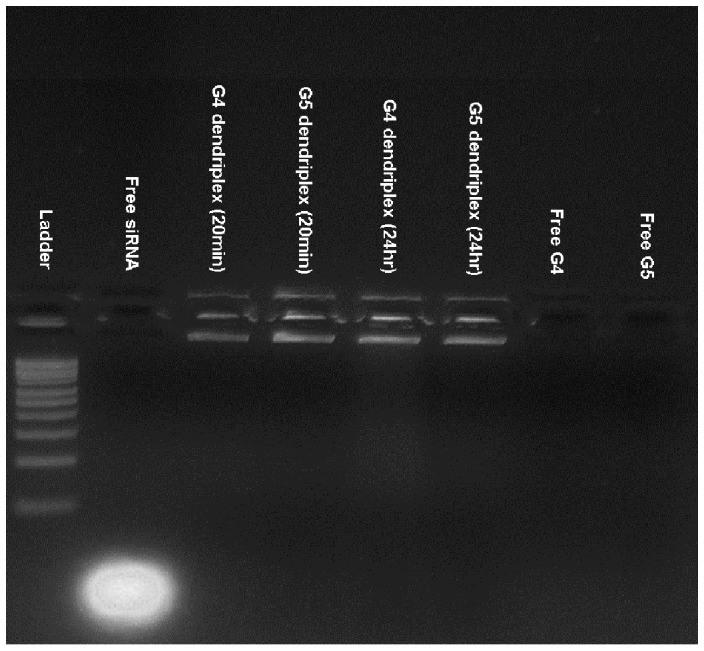
Electrophoretic mobility of free siRNA, free G4 and G5 dendrimers, and the particles. Image of the 1% w/v agarose gel stained with ethidium bromide showing the electrophoretic mobility of free siRNA, free G4 and G5 dendrimers, and the particles prepared by mixing G4 and G5 dendrimers with 0.7 µg of anti-GAPDH siRNA at an N/P (+/−) ratio of 2/1 for 20 minutes or 24 hours.

Both G4 and G5 dendrimers successfully complexed the loaded anti-GAPDH siRNA molecules (0.7 µg) at an N/P (+/−) ratio of 2/1 via the electrostatic interaction between the cationic amine groups (N) and the anionic phosphate groups (P). The gel image shows that siRNA molecules were retained in the wells after mixing with G4 and G5 dendrimers for 20 minutes and 24 hours indicating rapid condensation of the loaded siRNA molecules by the cationic carriers (**Figure 1**).

### Effect of RNase enzyme on free siRNA

**Figure 2:**
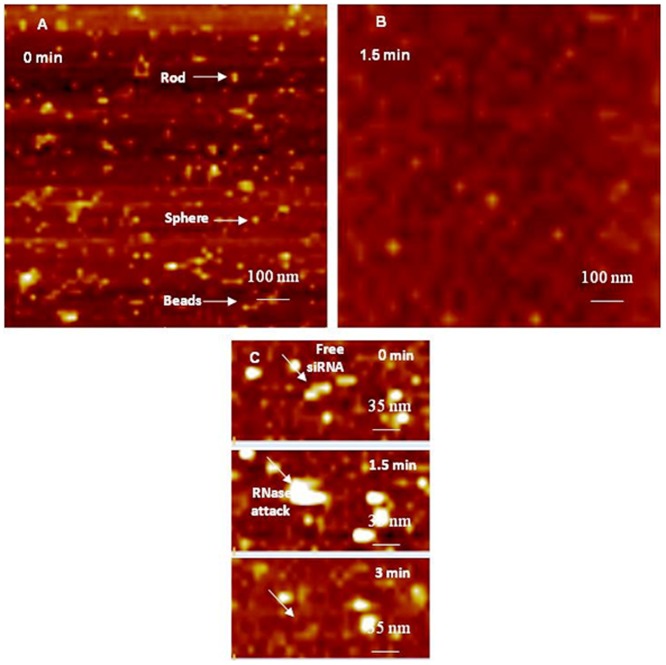
AFM image of free siRNAs before and after attack by RNase enzyme. (A) AFM image of free anti-GAPDH siRNA dissolved in 1 mM PBS containing 2 mM MgCl_2_ after adding to the surface of freshly cleaved mica, which shows rod-, sphere-, and bead-like arrangements. (B) AFM image taken 1.5 minutes after adding RNase V1 enzyme, which shows rapid fragmentation of adsorbed siRNA molecules. (C) Time-lapse images showing a single siRNA molecule denoted by the white arrow (*t* = 0 min), the attack of RNase V1 enzyme on free siRNA molecule (*t* = 1.5 min), and complete siRNA degradation (*t* = 3 min). The scale bar in images A and B is 100 nm and the Z scale is 9 nm. The scale bar in image C is 35 nm and Z scale is 7 nm.

Atomic Force Microscopy (AFM) is a powerful surface analytical technique that is routinely used to image and characterizes objects in the sub nanometer scale [34]. One key advantage of AFM compared to other high resolution microscopy techniques is simple sample preparation that does not involve negative staining or shadow casting with a metal coating, which allows direct measurements that reflect the natural surface of the specimen [35], [36], [37]. AFM uses piezoelectric ceramics to move a probe mounted on a cantilever in sub nanometer scale increments in the X, Y, and Z directions. The probe is positioned fractions of a nanometer above the sample of interest to track its topography at a constant force, which allows the cantilever to experience the smallest attraction and repulsion forces in sub nano Newton scale (nN) that bends the cantilever due to topography changes. The cantilever movement is tracked by a laser beam that reflects off the cantilever to a 4 chamber photo detector, which monitors and processes the deflection of the laser through a feedback loop. These deflections are processed into a 3-dimensional (3D) image using specific AFM software. In late 1980s, AFM was used to reproducibly image DNA and crystals of membrane proteins [38]. AFM was later used to image samples in liquid environment, which eliminated the interference of capillary and Van der Waals forces and allowed the imaging of biological events in a setting similar to the physiological environment [39], [40]. We capitalized on established AFM protocols to image free siRNA molecules, G4 and G5 dendriplexes in liquid, and their susceptibility to enzymatic attack. AFM images show that free siRNA molecules added to freshly cleaved mica appear as short rods, spheres, or threaded beads (**Figure 2A**). The average diameter of siRNA spheres is 18.3±3.1 nm while the average length of the rod- and bead-like features is 12.1±0.6 and 51±12.4 nm, respectively. These rod-, sphere-, and bead-like particles are probably formed via electrostatic interaction between cationic Mg^+2^ ions and multiple anionic siRNA molecules [25]. AFM images show that treatment of free siRNA molecules adsorbed to mica surface with RNase V1 enzyme results in their fragmentation within 1.5 minutes due to rapid and unrestricted access of the enzyme to RNA surface (**Figure 2B**). Time-lapse images clearly show individual siRNA molecule adsorbed to mica surface denoted by the white arrow before the addition of RNase V1 enzyme (i.e. *t* = 0 minutes) (**Figure 2C**). RNase V1 enzyme was visualized after 1.5 minutes of adding the enzyme solution to free siRNA, which caused complete degradation of adsorbed siRNA molecule within 3 minutes (**Figure 2C**). These images clearly show rapid accessibility of RNase V1 enzyme to free siRNA molecules leading to their degradation, which is consistent with earlier results [25], [41].

### Effect of RNase enzyme on G4 dendriplexes

**Figure 3:**
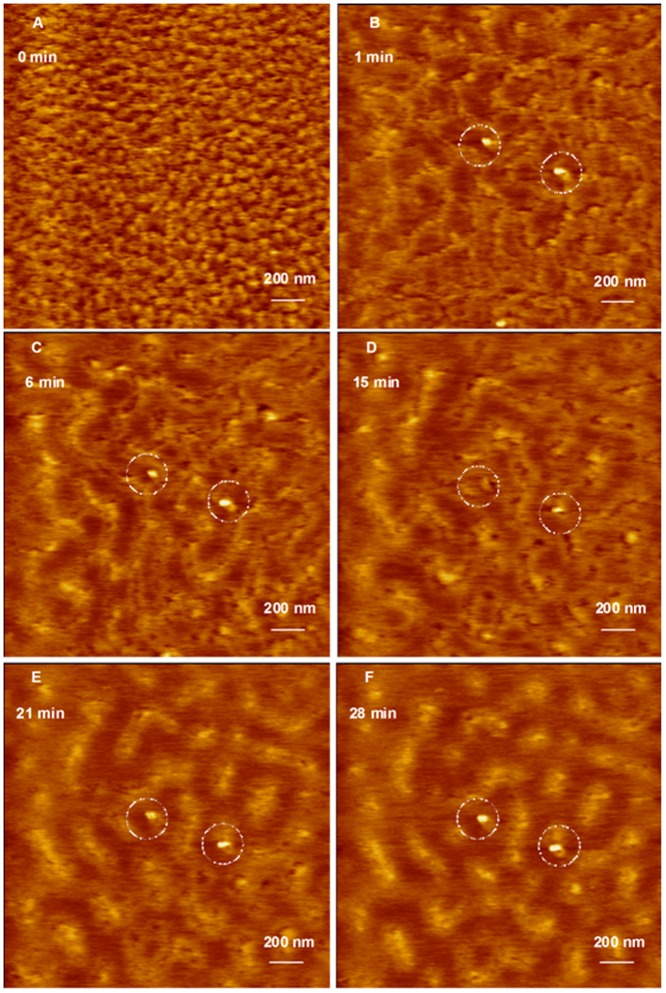
AFM image of G4 dendriplexes prepared for 20 minutes attacked by RNase enzyme. (A) AFM image of hexagonal G4 dendriplexes prepared by mixing of G4 dendrimers with 0.7 µg of anti-GAPDH siRNA at N/P ratio of 2/1 for 20 minutes at room temperature before loading onto the surface of freshly cleaved mica. AFM images of G4 dendriplexes after incubation with RNase V1 enzyme for 1–28 minutes (B–F) shows separation of the adsorbed dendriplexes and degradation of the complexed siRNA molecules (dark spots) that increased with the increase in incubation time. Two dendriplexes (defined wit dotted circles) remained intact throughout the incubation time with RNase V1 enzyme suggesting the formation of individual compact particles. Scale bar in these images is 200 nm and the Z scale is 15 nm.

Incubation of G4 dendrimers with anti-GAPDH at an N/P ratio of 2/1 for 20 minutes yielded dendriplexes that were visualized using AFM (**Figure 3**). AFM images show that G4 dendriplexes are compact hexagonal particles with an average full width of 43±19.3 nm at half the maximum height and an average height of 0.63±0.025 nm (**Figure 3A**). Our preliminary studies indicated the difficulty in imaging individual G4 dendriplexes and individual siRNA fragments released upon incubation with RNase V1 enzyme at different time points. Therefore, we investigated the effect of RNase V1 enzyme on a monolayer of G4 dendriplexes covering an entire AFM imaging field before and after enzyme addition instead of trying to image an individual particle. AFM images show that G4 dendriplexes formed a densely packed monolayer on freshly cleaved mica surface before the addition of RNase V1 enzyme (**Figure 3A**). However, addition of RNase V1 enzyme separated the imaging field into bright spots where intact hexagonal G4 dendriplexes were located and dark spots where the particles got detached and the complexed siRNA was degraded within 1 minute (**Figure 3B**). AFM images show that increasing the incubation time (6, 15, 21, and 28 minutes) with RNase enzyme increased siRNA degradation shown by the increase in the fraction of dark spots in the imaging field (**Figure 3C–3F**). **Figure 3B–F** show two large G4 dendriplexes marked by the dashed circles with heights of 9.4 and 7.6 nm and diameters of 151 and 110 nm from left to right. These complexes remained intact throughout the incubation time (28 minutes) with RNase V1 enzyme indicating better shielding of their RNA cargo compared to the bulk of G4 dendriplexes.

**Figure 4:**
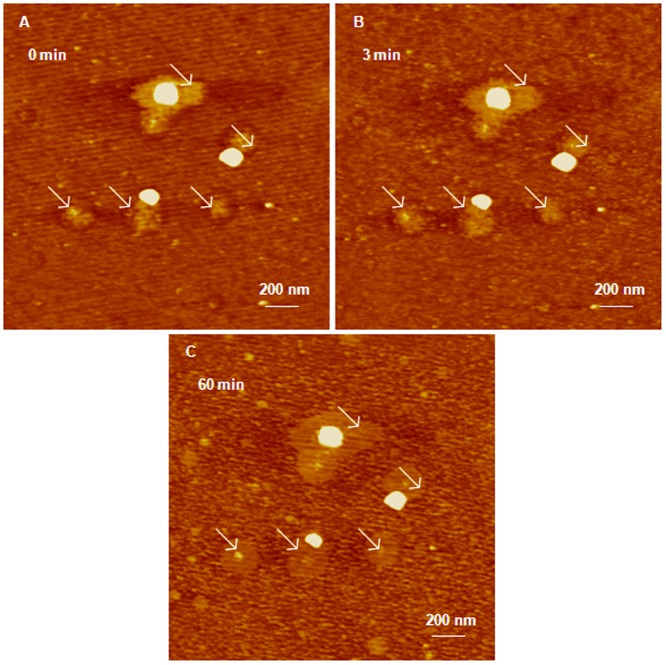
AFM image of G4 dendriplexes prepared for 24 hours attacked by RNase enzyme.

In comparison, G4 dendriplexes prepared by mixing of G4 dendrimers with anti-GAPDH siRNA at an N/P ratio of 2/1 for 24 hours appeared as large globular particles with a dense central core surrounded by “loose” network (**Figure 4**). AFM images show that the average diameter of the tightly-packed core is 263±60 nm with an average height of 35.3±8.6 nm (**Figure 4A**). Time-lapse AFM images show that incubation of these G4 dendriplexes with RNase V1 enzyme did not affect the compact central core for up to 60 minutes (**Figure 4B & C**). However, the network surrounding the core fragmented gradually with the increase in incubation time with RNase V1 enzyme indicated by the reduction in their height from 12.3±1.2 nm to 3.4±1.5 nm (**Figure 4B & C**). These results clearly show that increasing the incubation time of anti-GAPDH siRNA with G4 dendrimers from 20 minutes to 24 hours results in the formation of larger and more tightly-packed complexes that resist enzymatic degradation by RNase enzymes. These results are supported by previous studies indicating that complexation of siRNA molecules with G4 dendrimers is a biphasic process that starts with an initial exothermic binding process followed by secondary endothermic formation of larger dendriplex aggregates [42], [43].

### Effect of RNase enzyme on G5 dendriplexes

**Figure 5:**
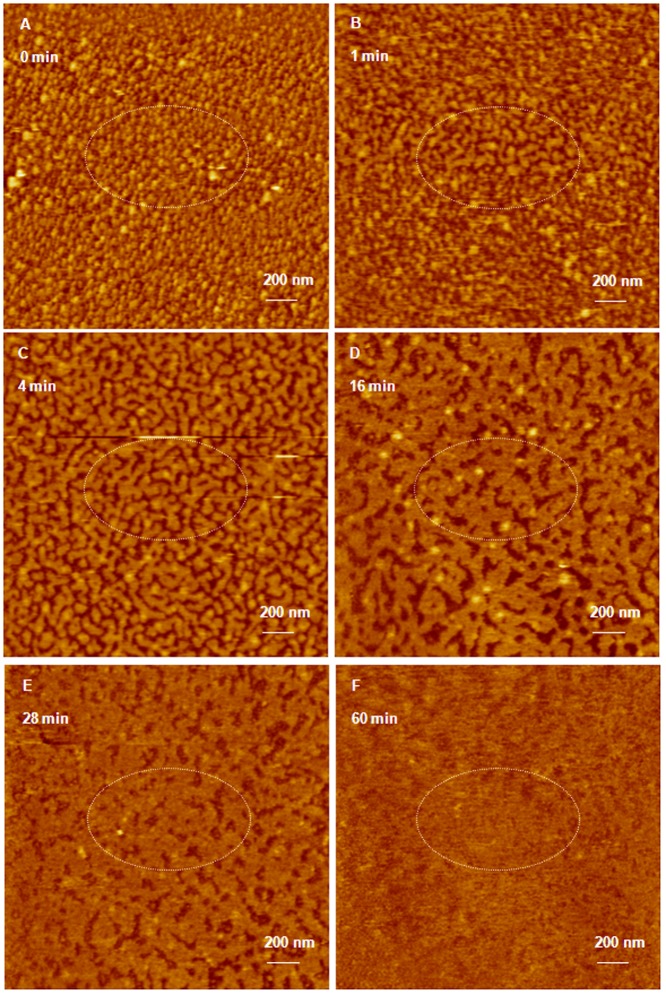
AFM image of G5 dendriplexes prepared for 20 minutes attacked by RNase enzyme. (A) AFM image of hexagonal G5 dendriplexes prepared by mixing of G5 dendrimers with 0.7 µg of anti-GAPDH siRNA at N/P ratio of 2/1 for 20 minutes at room temperature before loading onto the surface of freshly cleaved mica. AFM images of G5 dendriplexes after incubation with RNase V1 enzyme for 1–60 minutes (B–F) shows separation of the adsorbed dendriplexes and degradation of the complexed siRNA molecules (dark spots) that increased with the increase in incubation time. Scale bar in these images is 200 nm and the Z scale is 17 nm.

**Figure 6:**
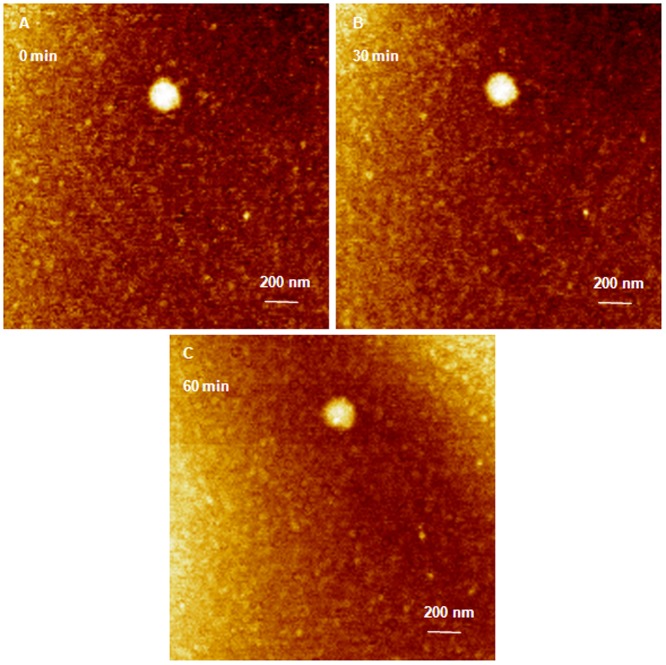
AFM image of G5 dendriplexes prepared for 24 hours attacked by RNase enzyme. (A) AFM image of G5 dendriplexes prepared by mixing of G5 dendrimers with 0.7 µg of anti-GAPDH siRNA at N/P ratio of 2/1 for 24 hours at room temperature before loading onto the surface of freshly cleaved mica. G5 dendriplexes remain intact upon incubating with RNase V1 enzyme for 30 (B) and 60 minutes (C). Scale bar in these AFM images is 140 nm and the Z scale is 5 nm.

Similarly, incubation of G5 dendrimers with anti-GAPDH at an N/P ratio of 2/1 for 20 minutes yielded compact hexagonal dendriplexes that formed a densely packed monolayers on the surface of freshly cleaved mica with an average full width of 62±8.3 nm at half the maximum height and an average height of 1.1±0.05 nm (**Figure 5A**). Addition of RNase V1 enzyme to the dendriplexes monolayer separated the imaging field into bright spots where intact hexagonal G5 complexes were located and dark spots where the particles got detached and the complexed siRNA was degraded (**Figure 5B–5F**). AFM images show that increasing the incubation time (1, 4, and 16 minutes) with RNase enzyme increased siRNA degradation shown by the increase in the fraction of dark spots in the imaging field (**Figure 5B–D**). Increasing the incubation time with the enzyme to 28 and 60 minutes allowed RNA fragments to fill the dark spots as shown in **Figure 5E–5F**. Increasing G5 incubation time with anti-GAPDH siRNA to 24 hours increased the width of the formed complexes to 48.3±2.5 nm at half the maximum height and the average height to 2.1±0.2 nm (**Figure 6A**). Despite of the smaller size of G5 dendriplexes, they remained intact upon incubation with RNase V1 enzyme for up to 60 minutes (**Figure 6B & C**) confirming that longer incubation time results in formation of tightly packed and more stable complexes.

## Discussion

Particles prepared by ionic complexation of PAMAM dendrimers with siRNA molecules were examined using AFM under air-dry conditions [41]. However, it is critical to evaluate the morphology of such complexes under physiologically-relevant conditions, which will impact complex stability against enzymatic attack, interaction with different cells, internalization mechanism, and overall transfection capacity. Therefore, we decided to visualize naked siRNA and G4 and G5 dendriplexes in solution using AFM, which will eliminate the destructive effect observed upon imaging biological samples in air [25], [40], [44]. Imaging naked siRNA or G4 and G5 dendriplexes in solution in presence or absence of RNase V1 enzyme proved challenging due to free mobility of these particles in solution. To address this issue, we used small divalent Mg^+2^ cations to allow weak electrostatic adsorption of free siRNA and G4/G5 particles to mica's surface following previously published protocols [33]. We applied weak forces (pico Newtons) by the tip of the AFM imaging probe to avoid delocalizing the examined samples coupled with changing the X and Y offsets as needed to track the moving features within the imaging field as previously reported [25], [45]. We also used a fast scanning speed (90 seconds/image) to avoid repelling the imaging tip by the examined sample [25], [45]. These settings allowed us to visualize free siRNA molecules, G4 and G5 dendriplexes, and the attack by RNase V1 enzyme in solution.

AFM images show that despite the difference in size, number of cationic amine groups, and flexibility of G4 and G5 dendrimers, they formed similar hexagonal particles when incubated for 20 minutes with anti-GAPDH siRNA (**Figures 3A** **&** **5A**). Increasing the incubation time of G4 and G5 dendrimers with anti-GAPDH siRNA to 24 hours produced larger dendriplexes compared to those observed at shorter incubation time point (**Figures 4A** **&** **6A**). However, G5 formed smaller dendriplexes compared to G4, which can be explained by the fact that G5 has twice the number of cationic amine groups (+) compared to G4. Therefore, the number of G5 particles used to complex 0.7 µg of anti-GAPDH siRNA is half the number of G4 particles used to complex the same amount of RNA. The relatively smaller number of G5 particles coupled with their established rigidity compared to G4 makes it harder for siRNA molecules to form intra-molecular bridges between multiple G5 particles via electrostatic interaction, which reduced the size of the formed dendriplexes. This is further confirmed by the absence of the loose network observed with G4 dendriplexes (**Figure 4A**). These images indicate that complexation of anti-GAPDH siRNA to G4 and G5 dendrimers is a biphasic process that starts with a rapid exothermic binding forming “loose” dendriplexes followed by a slow endothermic formation of highly compacted complexes similar to previous reports [25], [27], [42], [43], [46], [47]. Further, the observed rapid degradation of G4 and G5 dendriplexes prepared within a short incubation time (20 minutes) indicates the G4 and G5 dendrimers could not “shield” the cleavage sites of the complexed RNA molecules from the RNase V1 enzyme (**Figures 3B–D** **&** **5B–F**). In comparison, formation of compact particles at longer incubation time proved effective in shielding and protecting the loaded RNA from the attack of RNase V1 enzyme (**Figures 4** **&** **6**).

## Conclusions

AFM images show that increasing the incubation time of G4 and G5 dendrimers with siRNA molecules to 24 hours results in formation of ionic complexes that can protect the loaded RNA cargo against enzymatic degradation without using excess cationic dendrimers. Further, the size of the formed complexes can be tuned by controlling the flexibility of the cationic carrier with flexible G4 forming larger particles than the more rigid G5 dendrimers while maintaining the desired enzymatic stability. These findings provide insight on potential formulation strategies to develop enzymatically-resistant complexes with tunable size for enhanced intracellular delivery of therapeutic siRNA molecules without inducing undesirable side effects due to the use of excess cationic carrier.
